# Family involvement in advance care planning for people living with advanced cancer: A systematic mixed-methods review

**DOI:** 10.1177/02692163211068282

**Published:** 2022-01-06

**Authors:** Megumi Kishino, Clare Ellis-Smith, Oladayo Afolabi, Jonathan Koffman

**Affiliations:** Cicely Saunders Institute of Palliative Care, Policy & Rehabilitation, Florence Nightingale Faculty of Nursing, Midwifery & Palliative Care, King’s College London, London, UK

**Keywords:** Advance care planning, neoplasms, family, systematic review

## Abstract

**Background:**

Advance care planning is important for people with advanced cancer. Family involvement in advance care planning may be instrumental to achieving goal-concordant care since they frequently become surrogate decision-makers.

**Aim:**

To examine components, contexts, effects and linkages with intended outcomes of involving family members in advance care planning.

**Design:**

A mixed-methods systematic review, in which quantitative and qualitative data were extracted and synthesised using thematic synthesis leading to a logic model. Prospectively registered on PROSPERO (CRD42020208143).

**Data sources:**

Primary quantitative and qualitative research regarding family-involved advance care planning for people with advanced cancer were identified using Medline, Embase, PsycINFO and CINAHL from inception to September 2020. Quality appraisal was performed with ‘QualSyst’.

**Results:**

Fourteen articles were included. The synthesis identified perceptions of individuals and family members concerning family involvement in advance care planning and presents components for family-integrated advance care planning intervention. The logic model includes (i) addressing family members’ concerns and emotions and (ii) facilitating communication between individuals and family members which are distinctive when healthcare professionals engage with individuals as well as family members.

**Conclusions:**

This review provides a comprehensive understanding of family involvement in advance care planning and could inform its assessment and implementation in clinical practice. The number of included articles was limited. Therefore future research must focus on family integration and exploration of stakeholders’ perceptions to identify additional components and linkages between them within family-integrated advance care planning.

**What is already known about the topic?**

- Advance care planning improves goal-concordant care.
- Family involvement in advance care planning is important to achieve this goal, however, this is frequently challenging and complex.
- There have been no attempts to systematically synthesise findings concerning family involvement in advance care planning.

**What this paper adds?**

- People with advanced cancer wish to involve family members in advance care planning if it benefits family members but may be concerned about engaging them in a potentially emotionally laden process.
- A logic model was developed in this review that includes two distinctive components: to assess and address family members’ concerns and emotions and to facilitate communication between individuals and their family members.

**Implications for practice, theory or policy**

- Engaging with family members during advance care planning discussions may indirectly motivate individuals to have advance care planning discussions and increase the likelihood of further dialogue between individuals and family members likely to occur.
- Future research should place more focus on family involvement in advance care planning to further refine the logic model to inform a family-integrated advance care planning intervention.

## Background

Cancer is the leading cause of death globally. Numbers are projected to rise from 9.96 million in 2020 to over 16.3 million in 2040.^1^ Of those with advanced cancer, rapid deterioration and unexpected deaths occur in up to 22% of individuals.^2,3^ Certain cancers, for example, brain tumours or metastases, impact the ability of individuals to make decisions before their condition further deteriorates.^4^ Consequently, communicating preferences about future treatment and care to family members and healthcare professionals before losing mental capacity is vital to align treatment and care with the individual’s wishes.

Advance care planning is a process that enables individuals to identify values, goals and preferences for future medical treatment and care, and to discuss these with family members and healthcare professionals. These preferences are recorded and reviewed so that they can be taken into account when individuals are no longer able to make decisions for themselves.^5^ Most recently, advance care planning has been widely endorsed during the COVID-19 pandemic as a means of facilitating people’s preferences regarding future care under challenging conditions.^6–8^ There is evidence advance care planning can improve concordance between preferences for care and delivered care, completion of advance directives, end-of-life care discussions and satisfaction with care.^9–11^ Advance care planning also contributes to reducing stress, anxiety and depression in bereaved family members.^10^ Moreover, discussing wishes about care at the end-of-life can result in lower healthcare costs and better quality of life with no difference in survival compared to those without advance care planning.^12^

Family members are often required to be ‘surrogate’ decision-makers when their relatives lose the capacity to make decisions for themselves.^13^ However, there is evidence that surrogates may incorrectly predict their relatives’ end-of-life treatment preferences^14^ and that they may experience emotional distress when engaging in treatment decisions on their relatives’ behalf.^15^ Family involvement in advance care planning or shared decision making represents ‘*a process in which decisions are made in a collaborative way, where trustworthy information is provided in accessible formats about a set of options, typically in situations where the concerns, personal circumstances, and contexts of patients and their families play a major role in decisions*’.^16^ There are at least two potential benefits associated with family involvement. First, communication sharing between individuals and their family members enables family members to become aware of their relatives’ wishes and how they might change over time. This may contribute to goal-concordant care and a commensurate reduction of surrogate decision-making burden in family members.^17,18^ Second, family members may enable and empower their relatives to identify values, goals and preferences that matter to them.^19^ However, findings regarding family involvement in advance care planning suggest it is challenging and complex.^20^ Moreover, there has been no attempt to systematically synthesise the data in family involvement in advance care planning. This study therefore aims to systematically identify, appraise and synthesise existing evidence to inform the development of a logic model of family-integrated advance care planning for people with advanced cancer. Specifically, it examines the experiences and perceptions of people with advanced cancer and their family members concerning advance care planning and examines the components, contexts, direct and intermediate effects and linkages with advance care planning’s intended outcomes.

## Methods

The research paradigm underpinning this systematic review is represented by pragmatism.^21,22^ This approach is based on the proposition that researchers should use the philosophical and/or methodological approach that works best for the particular research problem that is being investigated. It is here that ‘pragmatist researchers’ consider the research question to be more important than either the methods they use or the paradigms that underlie the methods.^23^ Moreover, Maxcy suggests that pragmatism emerges as both a method of inquiry and a device for the settling of battles between research purists and more practical-minded scientists.^24^ We adhere to this sentiment and rationalise the conjoint use of quantitative and qualitative methods as being complementary and necessary to address the overarching aim of this review leading to the development of a logic model.^25^ Consequently, we see dual value in the qualitative research that explores in detail participants’ experiences and perceptions whereas the quantitative research used in this review examines the relationship between the components. The Preferred Reporting Items for Systematic reviews and Meta-Analyses (PRISMA) statement^26^ and Enhancing transparency in reporting the synthesis of qualitative research (ENTREQ) statement^27^ guided the reporting. Details of the protocol for this systematic review were registered on PROSPERO (www.crd.york.ac.uk/PROSPERO/display_record.asp?ID=CRD42020208143).

### Search strategy and eligibility criteria

The literature search was conducted using the following electronic databases: MEDLINE, EMBASE, CINAHL and PsycINFO from their inception to 22 September 2020. We limited language to English. Additional search strategies included reviewing references of included articles and those key studies as well as the citations included textbooks, reports, and websites by using Web of Science. In addition, the following journals published during the last 5 years were hand-searched: *Journal of Pain and Symptom Management, Palliative Medicine, Psycho-Oncology, Journal of Palliative Medicine* and *BMJ Supportive & Palliative Care*.

The SPIDER framework was applied to inform keywords and study inclusion criteria. In addition, previous systematic reviews on advance care planning^9,11,20^ were utilised to refine the search strategy (Supplemental Table 1).

#### Sample

People with advanced cancer (which for this review advanced cancer refers to those with an incurable or poor prognosis for example stage 4, metastatic cancer, stage 3 with the physicians’ estimation of a possibility of 1-year mortality^28^ or in specific primary sites including pancreas, hepatobiliary, and lung which have low 5-year survival rates) and who have decisional capacity and their family members (aged 18-years or older)

#### The phenomenon of interest

Advance care planning with family presence during an advance care planning intervention or discussion with individuals. Advance care planning is either or combination of the following, through discussion between individuals and healthcare professionals, or family members concerning: (i) the identification of values and defining goals and preferences for future medical treatment and care, (ii) the identification of a personal representative and the recording of preferences.

#### Design

All types of research design.

#### Evaluation

Since outcomes of advance care planning vary all individuals’ and family members’ outcomes were included.

#### Research type

All types of primary studies and quality improvement studies. Experimental studies, quasi-experimental studies and observational studies that examine advance care planning intervention with family involvement among people with advanced cancer. Qualitative studies and mixed-methods studies that explored the experiences and perceptions of advance care planning that involve family members of people with advanced cancer, their family members and healthcare professionals. Studies were excluded if:

1. The number of study participants with advanced cancer accounted for less than 50% of the study sample.
2. Advance care planning was part of a more comprehensive intervention e.g. palliative care consultation.
3. Advance care planning was defined as comprising only ‘documentation of preferences’ and that the appointment of a personal representative with discussion was not between an individual with advanced cancer and healthcare professionals or the individual with advanced cancer and their family.
4. Original data were not present or were deemed to be insufficient for example protocol only or conference abstract.
5. Solely focussed on clinician outcomes.

### Study selection

The titles and abstracts of searched articles were screened by one reviewer (MK) to ensure they matched the inclusion criteria. If there was any uncertainty, the full text was obtained and examined in detail. Additional information was sought from study authors where necessary to judge exclusion. Reservations regarding exclusion were resolved through discussion with other reviewers (CE-S, OA and JK) where necessary. Finally, the second independent reviewer (OA) randomly confirmed 10% of included articles as to whether they matched the inclusion criteria.

### Quality appraisal

All included reports were appraised using QualSyst tools as it is suitable for both quantitative and qualitative studies.^29^ Based on previous studies using QualSyst^20,30,31^ a summary score was used to provide an overall measure of quality where scores of >80% were judged as ‘strong’, 71%–80% as ‘good’, 51%–70% as ‘adequate’ or <51% as ‘limited’. One reviewer (MK) assessed the quality of all included published articles. Half of all included studies were then randomly selected and independently scrutinised by a second reviewer (OA). Any disagreements were resolved by discussions with JK and CE-S.

### Data extraction

A data extraction sheet was developed and piloted. Text, tables and figures under the headings of ‘methods’, ‘results’ and ‘discussions’ were extracted. Data regarding the details of included quantitative studies were extracted using the Cochrane Consumers and Communication Group Data extraction template for included studies.^32^ Data regarding advance care planning interventions were extracted based on the template for intervention description and replication checklist and guide.^33^ Data regarding the details of included qualitative studies were extracted based on standards for reporting qualitative research (SRQR).^34^ Items included the intervention procedure (e.g. how and who provided the advance care planning intervention), the experiences and perceptions of individuals, family members, and healthcare professionals regarding family involvement in advance care planning (e.g. the answers of the questionnaire and their quotations in the interview) as well as general information (e.g. the title, aim and design). Data extraction was conducted by one reviewer (MK) with verification of extracted data from 10% of the included articles by a second reviewer (OA). JK and CE-S provided oversight for this process.

### Data synthesis

We used a data-based convergent design in which quantitative and qualitative data from all included articles were analysed using the same synthesis method after data transformation (e.g. quantitative data is transformed into themes) and the results are presented together.^35^ Specifically, thematic synthesis was performed to explore family involvement in advance care planning and included (i) the experiences and perceptions of people with advanced cancer and their family members and (ii) components of the advance care planning interventions, their direct and intermediate effects and linkages with intended outcomes. We selected thematic synthesis since it permitted us to reflect on the findings from primary studies, transparently synthesising them to generate an additional understanding.^36^ One reviewer (MK) conducted line-by-line coding of the findings of primary studies. This was followed by constructing ‘descriptive themes’ inductively, then ‘analytical themes’ that addressed our review questions were developed. A second reviewer (OA) engaged in a process of iterative discussion with the reviewer (MK) and JK and CE-S verified the data synthesis. These themes were used to construct a logic model referring to the guidance by Rohwer.^37^

## Results

### Search results

**Figure 1.**
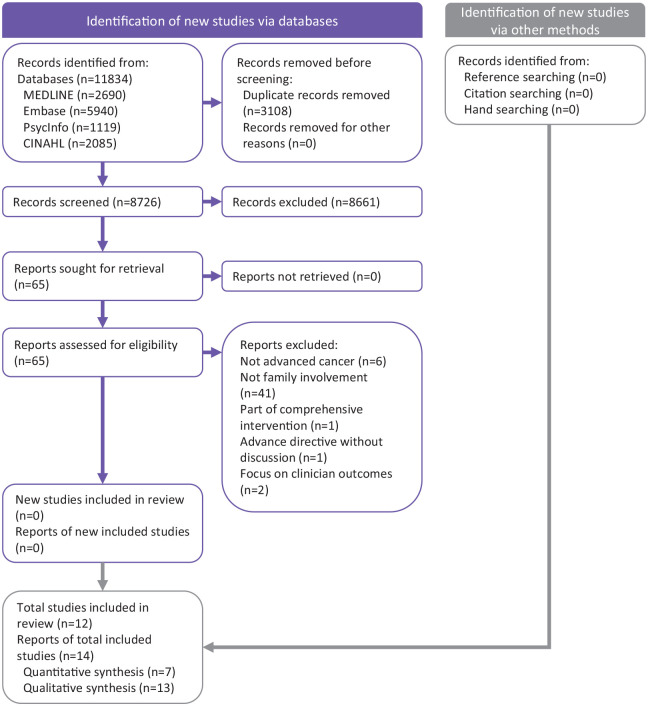
PRISMA flow diagram of the study inclusion process.

In total, 11,843 study abstracts were identified of which 14 articles were included. Figure 1 presents the PRISMA flow chart of this systematic review.

### Study characteristics

The description of included articles is presented in Table 1 for qualitative data and Table 2 for quantitative data. All articles were published after 2012. Five quantitative studies^17,38–41^, seven qualitative studies,^42–48^ a mixed-methods study^49^ and a quality improvement study.^50^ Six studies were conducted in the USA^38,44,45,47,48,50^, five in Australia^17,39,40,43,46^, two in Taiwan^42,49^, one in China.^41^ No study explicitly examined the effectiveness of family involvement in advance care planning. Since family-related outcomes measured in the included articles varied a cross-study comparison is not possible.

**Table 1.** Description of included articles with qualitative data.

Author and country	Purpose or research question	Participants	Design	Data collection	Main findings	Quality (%)
Lin et al.,^42^ Taiwan	To explore the decision-making processes and drivers associated with receiving palliative care in advance care planning discussions from perspectives of people living with advanced cancer, their families and healthcare professionals.	15 patients, 15 family members and 15 healthcare professionals	A part of project adopting a sequential explanatory qualitative mixed- methods approach	Semi-structured interviews	Drivers to choose palliative care were the expectations to reduce physical suffering from treatment, to avoid being a burden and to contribute to families and society. Opinions from families were highly influential on palliative care opition.	95
Lin et al.,^49^ Taiwan	To examine the feasibility and acceptability of a culturally adapted advance care planning intervention.	10 patients, 10 family members and nine healthcare professionals	Mixed-methods study	Semi-structured qualitative interview	Key contextual moderator included resource constraints, family’s influence, financial and policy support, and a presumption for end of life care provision and surrogate decision-making.	90
Michael et al.,^43^ Australia	To extend understanding of how family members view advance care planning to promote shared decision making.	18 family members (of 17 patients as part of a pilot advance care planning programme)	Qualitative descriptive design with grounded theory overtones	Focus group interviews or individual semi-structured interviews	Family members and family dynamics influence advance care planning decisions and actualisation of future care plan. Promoting shared decision making and supporting families is recommended.	80
Geerse et al.,^44^ USA	To characterise the content and interactions of conversations between trained oncologists and their patients informed by a structured conversation guide.	25 patients (conversations) conducted by 16 oncologists participating in an Serious Illness Care Programme RCT	Prospective qualitative design	Conversations were guided by Serious Illness Conversation Guide and audio-recorded	Patients were open to discussing values and goals, and willing to articulate preferences regarding life-sustaining treatment. Oncologists have difficulty in disclosing a time-based prognosis and responding to patients’ emotions.	80
Sharma et al.,^45^ USA	To explore decision-making by patients and clinicians during inpatient goals-of-care discussions.	62 patients having a goals-of-care discussion with 51 unique clinician	Qualitative, observational study	Goals-of-care discussions were audio-recorded	Clinicians did not always complete each shared decision making stage and what component they missed varied according to what decision they made with patients.	80
Johnson et al.,^46^ Australia	To explore how patients and their family members value autonomy at the end of life and to understand how this may impact on the way they develop and act on EoL decisions and planning.	Five patients and six family members participating in an advance care planning RCT	Prospective qualitative design	Semi-structured interview	Participants regarded advance care planning to enhance end-of-life care by decreasing uncertainty, enhancing comfort, helping to achieve ‘the small things’, and helping the family know what to do rather than to protect patients’ rights to determine what happent to their bodies.	75
Kumar et al.,^50^ USA	To characterise the experiences and perceptions of patients engaging in serious illness conversations as part of routine oncology care in the setting of Serious Illness Care Programme implementation.	32 patients	Quality improvement study	Qualitative interviews	Serious illness conversations had a positive impact on prognositc understanding and end of life planning. Improvement in the delivery of prognosis and preparing patients for serious illness conversations were suggested.	75
Epstein et al.,^47^ USA	To understand, in detail, patients’ rationale for their CPR preferences, and their thoughts about the advance care planning process in general.	26 patients participating in an CPR information material RCT	Exploratory qualitative data analysis approach	The responces to an open-ended question were written down by study staff verbatim.	Patients were apprehensive about advance care planning but wanted to discuss it. CPR video education is appropriate and an affirming initiator of advance care planning discussions.	70
Robinson,^48^ USA	To explore how unrealistic hope actually influences patients and their families in advance care planning.	18 participants comprised patients diagnosed with advanced lung cancer and their family members	Prospective qualitative design	Initial structured interview and additional interveiws where possible.	Hope was complicated and multi-faceted, well-considered possibility, and resilient and persistent. Advance care planning did not affect hope and hope for cure did not interefere with planning for end-of-life.	65

RCT: randomised controlled trial; CPR: cardiopulmonary resuscitation.

**Table 2.** Description of included articles with quantitative data.

Author and country	Objectives or Hypotheses	Design	Participants	Intervention	Primary and Family-related Outcome	Findings	Quality (%)
Johnson et al.,^17^ Australia	Hypothesised that advance care planning would increase discussion and documentation of patient wishes for EoL care and increase perceived and actual compliance with patients’ EoL wishes, improve the quality of death, and result in less mental and physical distress in family members.	RCT	Intervention *n* = 84 dyads Control *n* = 63 dyads	Respecting Patient Choices model that incoporated offering optional information abouth likely life expectancy. A meeting prior to an oncologist visit by trained nurses.	**Family members’ perception that the patients’ EoL wishes were discussed, and complied with** **Patient-reported communication of EoL care wishes with family and family-reported satisfaction with discussion, care, death and their mental well-being**	**No significant difference in family-reported wishes known and complied with, satisfaction with discussion, care and death. Communication between patients and family members was increased. Intervention components include asessing family members’ readiness. Smaller improvement in mental well-being of bereaved family members in intervention group.**	96
Lin et al.,^49^ Taiwan	To examine the feasibility and acceptability of a culturally adapted advance care planning intervention.	Mixed-methods study	Patient *n* = 10 Family *n* = 10 Healthcare professionals *n* = 9	Information materials, video decision aids, and communication coaching for patients, advance care planning consultation by trained healthcare staff	Fidelity of the intrevention and **the participants experiences**	Implementing a culturally adapted advance care planning intervention is feasible and acceptable in Taiwan. **Family involvement was regarded as one important component of the culturally adapted advance care planning.**	94
Rodenbach (2017), USA	To investigate how the intervention affected the number and nature of topics brought up during an oncology office visit.	Secondary analysis of RCT	Intervention *n* = 84 Control *n* = 86	Coaching session using question prompt list booklet prior to oncologist visit by trained social workers	**Total number of questions asked during the office visit and question prompt list-related topics brought up**	**Significant increase in bringing up question prompt list-related topic by either patients or family members during oncology visit. Family members were involved if available.**	93
Walczak et al.,^40^ Australia	Hypothesised that participants receiving the intervention would ask more questions and express more cues for discussion during an oncology office visit.	RCT	Intervention *n* = 61 Control *n* = 49	Communication support programme using question prompt list booklet prior to oncologist visit by trained nurses	**The number of questions and cues for discussion** **Predictors of patient and family members information seeking actions during oncology consultations**	Counts of questions and cues were significantly higher for patients in the communication support programme group in some topics. **In other topics, the presence of family members in consultations was associated with more questions from patients. Oncologists encouraged question asking was associated with family members to ask more questions and cues.**	93
Kumar et al.,^50^ USA	To characterise the experiences and perceptions of patients engaging in serious illness conversations as part of routine oncology care in the setting of Serious Illness Care Programme implementation.	Quality improvement study	Patient *n* = 32	Serious Illness Care Programme. Serious illness conversations using a conversation guide by trained oncology clinicians.	Prognostic understanding, sense of control over medical decisions, closeness with clinician, and hopefulness about quality of life.	About half of the participants reported increased understanding of their future health, an increased sense of control over future medical decisions, increased closeness with their clinician and an increased hopefulness about quality of life. **Qualitative components contained data regarding family involvement in advance care planning.**	88
Vaccaro et al.,^39^ Australia	To develop a theoretically based fidelity audit tool for advance care planning and apply the fidelity tool to audiotaped advance care planning sessions from a RCT.	Exploratory substudy of an RCT	Patient *n* = 55 Family *n* = 50	Respecting Patient Choices model that incoporated offering optional information about likely life expectancy. A meeting prior to an oncologist visit by trained nurses.	Fidelity rating (content and quality) **including healthcare professionals acknowledge family, clarify family preferences, check family understanding and encourage family questions**	Overall, content fidelity was high, but quality depended on each item. **Family were well acknowledged (86%) and encouraged to ask questions (76%); their preferences and understanding were checked in 60% of the sessions.**	85
Kwok et al.,^41^ China	To identify common factors influencing AD completion and to examine the implementation of completed ADs	Retrospective analysis	*n* = 47	Engaging patients in discussion of AD	Factors associated with ad completion **including risk factor predicting complicated bereavement**	No significant difference concerning factors evaluated for predicting AD completion. Patients aged >60 were positively associated. **The presence of any risk factors predicting complicated bereavement in family was was associated with lower AD completion.**	64

EoL: end of life; RCT: randomised controlled trial; AD: advance directive.

• Data regarding family involvement in advance care planning were shown in bold.

### Quality appraisal results

The quality of the articles was appraised as ‘strong’,^17,38–40,42,49^ ‘good’,^43–46^ ‘adequate’^41,47,48^ and one article with a combination of ‘strong’ in the quantitative and ‘good’ in the qualitative component.^50^ No articles were excluded.

### Synthesis of qualitative and quantitative data

Only data relevant to family involvement in advance care planning was used to be synthesised.

### People with advanced cancer and their family members’ experiences and perceptions

**Figure 2.**
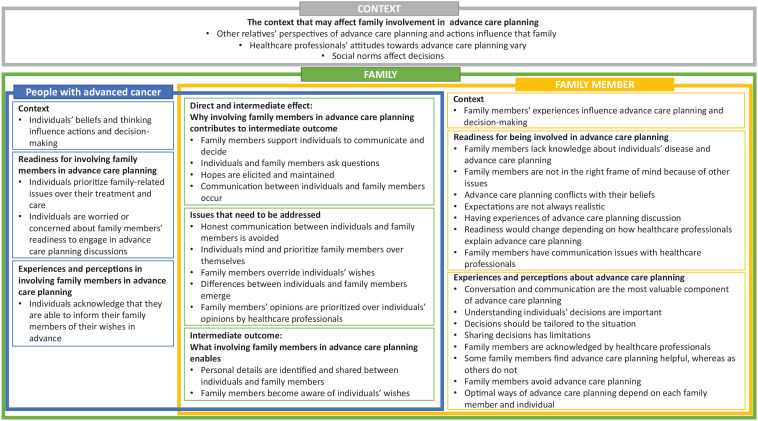
Individuals’ and family members’ experiences and perceptions of family involvement in ACP.

Figure 2 presents how the experiences and perceptions of people living with advanced cancer and their family members contribute towards the development of a model of family-integrated advance care planning. Specifically, this comprises four domains: perspectives of people with advanced cancer, perspectives of their family members, interactions within a family and the context outside of family.

#### Perspectives of individuals with advanced cancer

We identified various views concerning how individuals with advanced cancer view their family members. Some trusted their family members would act in their best interest.^46^ Others, however, did not wish to be a burden to their family members or wanted to maintain family harmony above their own interest. A 58-year-old female with cancer said, ‘*I will increasingly become a burden on my children rather than taking care of them if I have to live like this [lying in bed receiving life-sustaining treatment] and stay here [hospital]*’.^42^ These views consequently influenced their actions and decision-making.

Individuals had ambivalent opinions about family involvement in advance care planning. Some were motivated to commence advance care planning if they believed it would benefit their family members. Johnson et al. ^46^ observed individuals who were opposed to engaging in medical decision-making engaged in advance care planning discussions to protect family members from financial issues, ‘*Discussion of social world, relationships, grief, identity and the financial impact of death overwhelmed the data and formed participants’ largest concerns*’. However, individuals worried about their family members’ readiness to engage in this potentially emotionally laden process.^46,47^ This is supported by the findings from Kwok et al.^47^ who identified common factors that influenced advance directive completion; family members who were assessed at the risk of complicated bereavement were associated with lower advance directive completion.^41^ Understandably, individuals valued healthcare professionals support for family members during the process. This suggests that support for family members is indirectly for individuals. The perceived benefits of family involvement in advance care planning included individuals reporting they were able to inform their family members of their wishes in advance.^49^

#### Family members’ perspectives

The context in which family members experienced their relative’s disease trajectory influenced their attitudes towards advance care planning and decision-making. In some instances, when family members witnessed their relatives’ suffering first-hand this made them respect their decision to refuse future life-sustaining treatments for example, ‘*I accept it because I have looked after her [the patient] and seen how her suffering. I would only prolong [her death] if I insisted, she needed to receive the [life-sustaining] treatments*’.^49^

Family readiness to engage in advance care planning was also highly variable and depended on other factors. Some were not prepared because they lacked knowledge about individuals’ diseases or the potential benefits of advance care planning.^42,43,46,49^Others were not in the right frame of mind because they were overwhelmed with other issues.^43,46^ advance care planning was sometimes seen as challenging their beliefs.^43^ Some family members had already experienced advance care planning discussions before they had them with healthcare professionals^43^ or engaged in discussion with realistic expectations.^48^ Furthermore, others held out in hope of a cure for their relatives.^48^ For the latter, advance care planning discussions may be perceived by family members as negatively impacting their hope.

Healthcare professionals’ attitudes were also identified as influencing family members’ thoughts and actions. Where healthcare professionals emphasised the importance of advance care planning to them some family members similarly agreed.^47^ However, where problems communicating with healthcare professionals were present this made it difficult for family members to obtain adequate advance care planning-related information.^43^

Family members shared the positive and negative perceptions of advance care planning. They discovered that communication represented the most valuable component of the advance care planning experience.^46^ Through these discussions, they acknowledged that understanding an individual’s decision was central in coordinating their wishes.^43^ However, they also learnt the need to consider these decisions needed to flexibly accommodate future situations where necessary.^47^ There were also concerns that despite sharing wishes, these might not be followed up. This might account for why some family members found advance care planning helpful whereas others were more sceptical of its benefits. The former shared, ‘*advance care planning or participation in decision-making could be helpful in enhancing comfort, achieving “the small things”, and decreasing uncertainty*’.^46^ In contrast, the latter believed that wishes based on discussions about future decisions would not always be achieved and there was little point in discussing care in advance.^46^ A randomised controlled trial of the effectiveness of advance care planning discussion by trained facilitators demonstrated that there was no statistical significance in the proportion of family members who stated discussions with their relative helped to make decisions compared to those in the group of usual oncologist’s consultation (49% vs 45%, *p* = 0.76).^17^

In terms of healthcare professionals actions, family members emphasised the importance of assessment; the optimal ways depend on each family member and their relatives.^43,46^ Another important aspect of healthcare professionals actions related to how they engaged with not only individuals but also their family members during discussions. For example, healthcare professionals assessed family members’ readiness to engage in advance care planning.^17,49^ However, this was not always practised sufficiently. A fidelity study that examined the quality of healthcare professionals communication with family members during the advance care planning intervention identified healthcare professionals clarified family members’ preferences and understanding of their relatives’ disease in 60% of the sessions.^39^ Rodenbach et al.^38^ and Walczak et al.^40^ incorporated coaching sessions using instructional materials for individuals and their family members before discussions with oncologists. These researchers observed this information enabled two parties to consider topics important to them that would subsequently be discussed with their oncologists. In addition, Walczak et al.^40^ reported that oncologists endorsed question asking facilitated family members to ask questions and express a need for discussion.

#### Interactions within a family

Family interaction comprised three themes. The first explained why involving family members in advance care planning contributed to intermediate outcomes comprising four subthemes, for example, ‘family members support individuals to communicate and decide’ and ‘communication between individuals and family members occur’ (these components are used as ‘direct or intermediate effect’ in the following logic model). The second involved issues that healthcare professionals were required to address comprising five subthemes including ‘honest communication between individuals and family members is avoided’. The third explains what involving family members in advance care planning supports with two subthemes ‘personal details are identified and shared between individuals and family members’ and ‘family members become aware of individuals’ wishes’ (this is used as an ‘intermediate outcome’ in the logic model).

An important aspect about family involvement in advance care planning concerns that opportunities for dialogue between individuals and family members are created^49,50^ for example, ‘*My sons were with me, so it also gave pause for us to have a conversation. . .helped us make a game plan*’.^50^ Advance care planning intervention was associated with higher individual-reported communication of end-of-life care wishes with nominated family members.^17^ In addition, family members supported their relatives when making decisions.^43,48^ One study reported family members’ presence facilitated their relatives to ask questions and express their wish to discuss their concerns during hospital appointments with oncologists.^40^ Although one of the biggest issues healthcare professionals had was removing hope from individuals and their families by engaging in advance care planning discussions, Robinson^48^ observed that hope was paradoxically maintained in the process in which the participants were all dyads. This was achieved by engaging in a hypothetical possibility-related discussion that included ‘*hoping for the best, preparing for the worst*’ scenarios.

Several challenges were identified that need to be carefully considered by healthcare professionals. For example, despite individuals and family members caring deeply for one another communication can sometimes be stymied. This was manifest when honest communication between both parties was avoided. Specifically, family members may attempt to protect individuals from potential emotional distress by concealing their prognosis, and individuals become complicit in this by pretending they know nothing about.^42^ Second, individuals sometimes prioritised family members above themselves because they wanted to maintain harmony at their expense.^42^ Family members may, in some instances, overrule individuals’ wishes if they think it is in individuals’ or their families’ better interest. For example, a 38-year-old daughter said ‘he’ll say that he doesn’t want to be a burden . . . I’ll override all that’.^43^ These indicate that to share values, goals and preferences, healthcare professionals should facilitate communication between them based on the assessment of why their communication is hindered. However, it is possible that differences between them emerged because of facilitated communication. For example, a social worker said, ‘*If the treatment he expects is not the same as what the family expects, a conflict might arise*’.^49^ Moreover, in some cases, healthcare professionals prioritised family members’ positions over the individual’s, for example, healthcare professionals provided as many treatment options as were possible to avoid disputes with family members.^42^

Participants described what involving family members in advance care planning enabled that included two subthemes. First, participants described how they were able to share individuals’ preferences, goals and values. Specifically, it enabled individuals and family members to consider and then share personal details by expressing and understanding opinions.^47,49^ Second, through this process, family members became aware of their relative’s wishes typified by the following comment of a person with cancer, ‘*Well, I think there’s benefit many families for the communication and for understanding. Uhm . . . and for them to cope with your decision, I think. It’s a benefit to me and a benefit to them. I’m more relaxed about it and they are relaxed about it because there’s no difficult problems for them. It’s all done*’.^46^

#### Context outside of a family

Interactions between a family and their wider context were also described by participants. First, other relatives’ perspectives of advance care planning and their actions influenced the way decisions were made. For example, it may either intensify family members’ difficulties or alter their decision-making style. Michael et al.^43^ illustrates this by noting, *the family usually ‘collectively try and work something out’ when facing important decisions for example selling houses, but she [daughter] was now taking leadership on advance care planning because her brother says ‘it’s an awful topic’ and her sister was ‘kind of detached*’. Second, healthcare professionals’ attitudes towards advance care planning depended very much on the topic they were discussing and the person who was involved in that discussion. For example, some healthcare professionals only ever shared an individual’s prognosis with their family members and that family members’ requests were always prioritised.^42^ In addition, social norms influenced decisions, for example, those receiving palliative care were considered as having ‘given up’.^42^

### Intervention components relevant to family-integrated advance care planning

Table 3 presents the intervention components relevant to family-integrated advance care planning. Thirteen intervention components from five themes were identified. Assessing and understanding individuals and their family members was the fundamental part of the intervention to commence the advance care planning process. What healthcare professionals assessed and understood included individuals’ and family members’ understandings of their disease, understanding of advance care planning, their emotional state and their personal beliefs.^17,39,49^ Based on this assessment, healthcare professionals prepared individuals and family members for discussions about future care. For example, if they lacked knowledge about advance care planning healthcare professionals explained what it represented and the benefits it might confer.^49^ Identifying their concerns and questions and enhancing their communication ability were viewed as vital in empowering them.^38,40,49^ However, to permit family members to engage in discussions actively healthcare professionals were required to consciously reach out and address not only individuals’ but also family members’ concerns and emotions. This was demonstrated in a randomised controlled trial where the intervention group who engaged in advance care planning, and who comprised bereaved family members of individuals who had died of cancer, made only a small improvement from baseline (2.9) in their mental well-being as measured by the SF-12 compared to those who were not in receipt of the advance care planning intervention (change from baseline: 9.9), (*p* = 0.006). The authors indicated the possibility that the intervention adversely affected family members.^17^ Communication was central when sharing individuals’ values, goals and preferences. Healthcare professionals served to clarify family members understandings and preferences as well as the individuals.^39^ Not only did healthcare professionals facilitate communication between individuals and family members they also provided them with important opportunities to pause and reflect on their situation before making decisions.^50^ Moreover, healthcare professionals coordinated what was discussed and shared decisions in an appropriate way for example completed documentation which was copied to all parties.^49^

**Table 3.** The intervention components for family-integrated ACP.

Intervention components	Subcomponents	Procedures in each study
Assess and understand individuals and family members	Assess readiness	Assess the individual’s and/or family member’s readiness to discuss future care (e.g. timing, with or without family members, truth-telling, documentation)^12^
	Assess understanding	Assess the understanding of disease condition and prognosis from individual’ and families’ perspectives^28,37^
	Understand context	Understand patients’ and family members’ religion, belief and value of end of life care^37^
Prepare individuals and family members for a discussion about future treatment and care	Identify concerns	Provide Question Prompt List^27,29^
	Empower ability	Provide coaching session^27,29,37^
	Provide information	Provide informative materials (leaflets and video decision aids regarding advance care planning and life-sustaining treatment)^37^
		Explain the disease prognosis and ensure the individuals and family members understand the current disease condition^32,37^
**Discuss values, goals and preferences with addressing individuals and family members concerns and emotions**	Acknowledge family	Acknowledge family members^28,37^
		Demonstrate strong rapport by referring to family members^32^
	Create atmosphere	Endorse question asking to healthcare professionals^28,29^
		Secure sufficient time for consultation^29^
	Consider individuals’ and family members’ emotions	Approach with hypothetical possibilities in the context of ‘hoping for the best and preparing for the worst’^36^
		Ensure individuals’ comfort^32^
		Talk to family members about individuals’ prognosis^32^
**Facilitate communication between individuals and family members**	Clarify each view	Clarify each preference^28^
		Encourage questions^28,29^
	Let individuals and family members communicate	Give time for conversation between individuals and family members^38^
Coordinate what is discussed	Tailor decisions	Tailor an advance decision form for individuals with support from family members^37^
	Share the decisions	Keep the documents with the individual’s medical records and provide a copy for individual and family members^37^

•The following data regarding family involvement in advance care planning were synthesised: (i) the descriptions of intervention from the quantitative studies and (ii) the quotations of participants and the authors’ descriptions concerning what the individuals and their family members received and what healthcare professionals did in the advance care planning process from the qualitative studies.

•Distinctive components for a family-integrated advance care planning intervention were in bold.

## Discussion

This mixed-methods systematic review represents the first attempt to identify and synthesise evidence derived from different research approaches on how families can be involved in advance care planning for people with advanced cancer. Based on the syntheses of the experiences and perceptions of people with advanced cancer and their family members and the interventions in the articles this review conceptualises what a family-integrated advance care planning intervention may represent. The findings support the importance of engaging with both individuals and family members. Each family member, including a person with advanced cancer, influenced each other within a family^51^ as has been demonstrated in previous studies. For example, people with cancer and their family members locate treatment decision-making within the context of their relationships with others.^52^ Furthermore, any psychological distress present among family members may impact the person with cancer which in turn may then become amplified within the family.^53^ This review describes how this is also the case for advance care planning, suggesting that family members, the interaction within a family and the interaction between a family and wider context should also be considered concerning advance care planning practice.

### The logic model of family-integrated advance care planning

**Figure 3.**
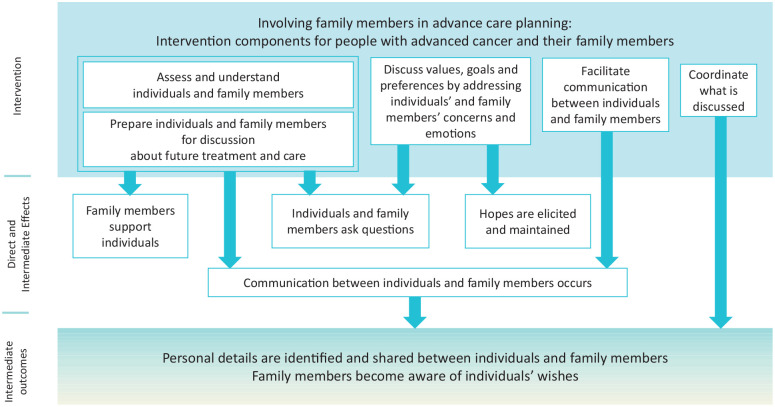
A logic model for family-integrated ACP intervention.

Figure 3 presents a logic model for family-integrated advance care planning intervention. The syntheses of qualitative and quantitative data identify and describe the individual components associated with family involvement in advance care planning that was subsequently used to develop the logic model. Specifically, the logic model comprises five intervention components, four direct and intermediate effects – what happens as a direct consequence of the intervention or its underlying processes that are associated with intermediate outcomes and two intermediate outcomes. This provides healthcare professionals and researchers with a comprehensive understanding of family involvement in advance care planning to enhance clinical practice and the further development of advance care planning interventions that involve families. The effects in the logic model were derived from qualitative data that were partially supported by quantitative data. Components that had no linkage found in this review with other components did not contribute to the logic model.

Of the intervention components, two distinctive components need to be highlighted that emerged owing to focussing on family participation in discussions. First, family members should be assessed and provided with support during interactions to address their concerns and emotions. This is because there will be those with advanced cancer who are willing to engage in advance care planning if they believe it also benefits their family members but do not want to cause them undue distress. Consequently, individuals valued the contribution of healthcare professionals in effectively and sensitively helping this occur. This is consistent with previous studies about the general public’s and palliative care population’s perspectives concerning advance care planning.^54,55^ Assessing and providing support for family members during discussion has the potential to enhance individuals’ willingness to progress the advance care planning process. This may also empower family members to help them cope with their respective issues, resulting in them regaining the power to support their relatives. Second, healthcare professionals may wish to facilitate communication between individuals and family members during discussion beyond healthcare professionals just becoming aware of individual’s values, goals, and preferences. This is because in many cases family members continue to share their relative’s disease trajectory until the end and that family members have a key role in informing healthcare professionals of the individual’s wishes on their behalf.

This review identified two important issues. First, communication is often avoided between the individuals and their family members and second, differences in wishes for future care arise between them. These concerns are evident concerning family involvement in cancer treatment decision-making during which individuals and family members view communication outside of the medical consultation as being beneficial.^56^ Advance care planning should therefore represent a continuation of that dialogue and the sharing of values. Healthcare professionals need to encourage and motivate both parties in having these discussions outside of healthcare professionals’ consultations. There is, however, some evidence that individuals who are well-educated or possess medical knowledge are less likely to involve family members in decisions about their cancer treatment^56^ and although accepting of advance care planning,^57^ their preference of family involvement in advance care planning remains unclear. However, advance care planning differs from cancer treatment decision-making in that it includes the consideration of decisions about future care in case of impaired decision-making capacity; the significance of family involvement in advance care planning lies not only in supporting individuals in decision-making but also ‘sharing personal details’ and ‘letting family members become aware of individuals’ wishes’. Therefore, the above results cannot be directly applied to advance care planning and it is necessary to examine what factors are associated with the preference for family involvement in advance care planning.

### Strengths and limitations of this review

The strength of this mixed-methods systematic review is that it attempted to include multiple realities – those from people with advanced cancer, their family members and healthcare professionals who care for them. This resulted in a deeper insight into this phenomenon. This review then synthesised the perspectives of these key stakeholders in the development of an initial logic model to guide the development of family-integrated advance care planning.

This study, however, has several limitations that limit the inferences that can be made from the findings presented. First, due to the comprehensiveness of our search strategy and the large number of records identified, primary screening was completed by one author. However, a second reviewer was brought in to scrutinise 10% of the extracted text at which point any differences in interpretation were discussed to reach a consensus. In addition, the overall research team were used to address unresolved concerns. Despite this, there remains a possibility of bias to the findings in the selection of studies and their interpretation.

Second, only English written articles were included from four countries, predominantly the USA and Australia where most advance care planning-related research appears to have taken place. This may also lead to a bias in the findings given that the nature of advance care planning and family involvement are linked to their respective cultures and law systems. However, we believe that reaching agreement among the reviewer team who represent various professional backgrounds and diverse cultural backgrounds may to some extent mitigate this concern.

Third, the descriptions of family involvement in advance care planning and the number of studies to support the linkages between the components are limited. Family members were involved in all included studies. For example, family involvement was considered as one important aspect of culturally adapted advance care planning intervention.^49^ However, only one study^43^ focussed explicitly on family involvement in advance care planning. The outcomes measured varied in the quantitative studies; two articles^39,49^ measured their intervention fidelity, two articles^38,40^ focussed on communication during discussion and one article^17^ examined the effectiveness for goal-concordant care and family-reported satisfaction of care and death and mental well-being. Only two studies^17,40^ were able to explain a linkage in the logic model. Therefore, this is a first step to reveal the complexity of family involvement in advance care planning and the initial logic model we have developed may have room for refinement.

### Implications for clinical practice

The interventions included in this review involved multi-professionals and in some instances over several consultations. Therefore, the intervention components do not necessarily have to be delivered in a single consultation to succeed. In addition, various professionals should play a role in this process that capitalises on their respective skills sets, expertise and the relationships they established with individuals and family members before the discussion. This aligns with the nature of advance care planning that is not a one-time event and that addresses individuals’ concerns across multi-dimensional aspects.^5^

### Implications for research

The logic model represents an initial model that requires further refinement. To realise this, the focus of research should be placed on family integration and exploration of stakeholders’ perceptions to identify additional components and linkages among them especially in countries other than the USA and Australia. This may inform the development of an effective family-integrated advance care planning intervention followed by examining its feasibility. Family involvement in advance care planning is complex and highly nuanced. Whether family involvement indeed contributes to goal-concordant care remains uncertain and may depend on multiple factors. Future research should attempt to examine these factors and their relative contribution to realising favourable outcomes. Factors may include, among others, individuals’ preferences of family involvement in advance care planning alongside the reasons and the characteristics of the population that would benefit from a family-integrated advance care planning intervention.

## Conclusions

The use of conceptual frameworks to underpin advance care planning delivery is presently uncommon. When used, frameworks typically focus on those living with life-limiting conditions rather than including their family members. This is surprising given that high-quality palliative and end-of-life care places particular emphasis on the family as being part of the equation of care. Despite this we identified, critically examined and synthesised evidence to develop an initial logic model of family-integrated advance care planning. Moreover, we describe how the components and processes of advance care planning operate using robust trial evidence from qualitative and quantitative studies. There are two distinct components in a family-integrated advance care planning logic model. First, healthcare professionals need to not only elicit the views of people with advanced cancer but also their family members. However, they need to be mindful that family members may have various issues other than advance care planning-related that are unrecognised or not addressed and which may influence the willingness to be involved. Furthermore, people with advanced cancer may well be concerned about their family members’ feelings and may require reassurance that healthcare professionals will support them. Therefore, addressing family members’ concerns and emotions may remove barriers to advance care planning that may, indirectly, improve advance care planning outcomes by empowering family members. Second, facilitating open communication between people with advanced cancer and their family members is critical. However, communication associated with advance care planning must be regarded as a continuum developed over some time. Healthcare professionals must therefore create an environment where communication can be facilitated not only with healthcare professionals but also between people with advanced cancer and their family members at this critical juncture in both their lives. Future research should explore additional components of family involvement in advance care planning and their linkages as well as developing and testing a family-integrated advance care planning intervention informed by the logic model.

## Supplemental Material

sj-pdf-1-pmj-10.1177_02692163211068282 – Supplemental material for Family involvement in advance care planning for people living with advanced cancer: A systematic mixed-methods review Click here for additional data file. Supplemental material, sj-pdf-1-pmj-10.1177_02692163211068282 for Family involvement in advance care planning for people living with advanced cancer: A systematic mixed-methods review by Megumi Kishino, Clare Ellis-Smith, Oladayo Afolabi and Jonathan Koffman in Palliative Medicine
