# Supplementary material for: Family involvement in advance care planning for people living with advanced cancer: A systematic mixed-methods review
Source: Palliat Med. 2022 Jan 6;36(3):462–77. doi: 10.1177/02692163211068282 (PMC8972955; doi:10.1177/02692163211068282)
Supplement: sj-pdf-1-pmj-10.1177_02692163211068282 – Supplemental material for Family involvement in advance care planning for people living with advanced cancer: A systematic mixed-methods review [file sj-pdf-1-pmj-10.1177_02692163211068282.pdf]

Supplementary Table 1 Search Strategy for Medline-Modified for Searches in Embase, PsycINFO, and CINAHL

1. exp Neoplasms/
2. exp Medical Oncology/
3. exp Psycho-Oncology/
4. Neoplasms.m_titl.
5. Neoplasms.mp.
6. limit 5 to abstracts
7. cancer.m_titl.
8. cancer.mp.
9. limit 8 to abstracts
10. Oncolog*.mp.
11. 1 or 2 or 3 or 4 or 5 or 6 or 7 or 8 or 9 or 10
12. "advanced cancer".mp.
13. "Incurable cancer".mp.
14. "Serious illness".mp.
15. "Life threatening".mp.
16. uncertainty.mp.
17. metastat*.mp.
18. 12 or 13 or 14 or 15 or 16 or 17
19. exp Advance Care Planning/
20. exp Family/
21. exp Caregivers/
22. exp Professional-Family Relations/
23. exp Communication/
24. advance care planning.m_titl.
25. advance care planning.mp.
26. limit 25 to abstracts
27. advance* care plan*.mp.
28. end of life discussion*.mp.
29. end of life care discussion*.mp.
30. "goals of care".mp.
31. "goal concordant care".mp.
32. surrogate decision make*.mp.
33. "surrogate decision making".mp.
34. "health care proxy".mp.
35. "family involvement".mp.
36. 19 or 20 or 21 or 22 or 23 or 24 or 25 or 26 or 27 or 28 or 29 or 30 or 31 or 32 or 33 or 34 or 35 or 36
37. 11 and 18 and 36
38. limit 37 to english language
